# Application of Orthobiologics in Achilles Tendinopathy: A Review

**DOI:** 10.3390/life12030399

**Published:** 2022-03-09

**Authors:** Luciano C. Ramires, Madhan Jeyaraman, Sathish Muthu, Navaladi Shankar A, Gabriel Silva Santos, Lucas Furtado da Fonseca, José Fábio Lana, Ramya Lakshmi Rajendran, Prakash Gangadaran, Manasi P. Jogalekar, Alfredo A. Cardoso, Alex Eickhoff

**Affiliations:** 1 Department of Orthopaedics and Sports Medicine, Centro Clínico Mãe de Deus, Porto Alegre 90110-270, Brazil; lucianoramires@gmail.com; 2 Department of Orthopaedics, Faculty of Medicine—Sri Lalithambigai Medical College and Hospital, Dr MGR Educational and Research Institute, Chennai 600095, India; madhanjeyaraman@gmail.com; 3 Department of Orthopaedics, Apollo Hospitals, Greams Road, Chennai 600006, India; navaladiortho@gmail.com; 4 Department of Orthopaedics, Government Medical College and Hospital, Dindigul 624304, India; 5 Department of Orthopaedics, The Bone and Cartilage Institute, Indaiatuba 13334-170, Brazil; ffonsecalu@gmail.com (L.F.d.F.); josefabiolana@gmail.com (J.F.L.); 6 Department of Orthopaedics, The Federal University of São Paulo, São Paulo 04024-002, Brazil; 7 Department of Nuclear Medicine, School of Medicine, Kyungpook National University, Daegu 41944, Korea; ramyag@knu.ac.kr; 8 BK21 FOUR KNU Convergence Educational Program of Biomedical Sciences for Creative Future Talents, Department of Biomedical Sciences, School of Medicine, Kyungpook National University, Daegu 41944, Korea; 9 Helen Diller Family Comprehensive Cancer Center, University of California San Francisco, San Francisco, CA 94158, USA; manasi.jogalekar@ucsf.edu; 10 Department of Oncology-Integrative Medicine-Pain Care, IAC—Instituto Ana Cardoso de Práticas Integrativas e Medicina Regenerative, Gramado 95670-000, Brazil; med.alfcardoso@gmail.com; 11 Department of Orthopaedics, Centro Ortopédico Eickhoff, Três de Maio 98910-000, Brazil; alexeickhoff@hotmail.com

**Keywords:** Achilles tendinopathy, orthobiologics, regenerative medicine

## Abstract

Orthobiologics are biological materials that are intended for the regeneration of bone, cartilage, and soft tissues. In this review, we discuss the application of orthobiologics in Achilles tendinopathy, more specifically. We explain the concepts and definitions of each orthobiologic and the literature regarding its use in tendon disorders. The biological potential of these materials can be harnessed and administered into injured tissues, particularly in areas where standard healing is disrupted, a typical feature of Achilles tendinopathy. These products contain a wide variety of cell populations, cytokines, and growth factors, which have been shown to modulate many other cells at local and distal sites in the body. Collectively, they can shift the state of escalated inflammation and degeneration to reestablish tissue homeostasis. The typical features of Achilles tendinopathy are failed healing responses, persistent inflammation, and predominant catabolic reactions. Therefore, the application of orthobiologic tools represents a viable solution, considering their demonstrated efficacy, safety, and relatively easy manipulation. Perhaps a synergistic approach regarding the combination of these orthobiologics may promote more significant clinical outcomes rather than individual application. Although numerous optimistic results have been registered in the literature, additional studies and clinical trials are still highly desired to further illuminate the clinical utility and efficacy of these therapeutic strategies in the management of tendinopathies.

## 1. Introduction

Achilles tendinopathy (AT) or tendinitis of the heel is one of the most common ankles and foot overuse injuries [1]. This musculoskeletal disorder usually affects professional and recreational athletes who engage in vigorous physical activities, such as jumping and running, but it may also develop in sedentary individuals [2]. Achilles tendon injuries are often quite devastating because, unlike some tissue types, tendons are poorly vascularized structures that rely upon synovial fluid diffusion to provide nutrition [3]. Instead of homologous tissue formation before the injured state, a healing tendon will form a fibrous scar tissue, which renders it mechanically weaker in comparison to the native tendon [4]. The strength deficit may increase the risk of further injury and complications. Treatment of AT with orthobiologics is designed to restore the original tissue properties of the tendon and reduce the susceptibility of secondary injuries and additional damage [5]. The management of tendon disorders has changed from reconstruction to regeneration. Tendon regeneration requires cellular components and ECM equilibrium, and mechanical loading [6]. The application of orthobiologics for the management of painful musculoskeletal disorders, namely AT, is still undergoing expansion in the literature and there is much to be discussed.

Despite the relative success with conservative measures, up to 30% of non-operative treated patients will fall back on symptoms [7]. Most of them receive different treatment approaches along with their condition, which impacts the healthcare system [8]. Trials and conventional studies do not evaluate the relative efficacy of all treatments, opening fields to different alternatives and defying physicians as to better decision making [9].

The objectives of this manuscript are as follows. Firstly, basic science and description of the pathological mechanisms undermining tendinopathies will be given. Secondly, potential treatment alternatives will be proposed by reviewing the current findings of various therapies employing the use of orthobiologics for the treatment of AT. This review also throws light on the scaffold-free cellular and acellular therapies as promising regenerative medicine tools for the management of AT.

## 2. Etiopathogenesis of AT

**Figure 1:**
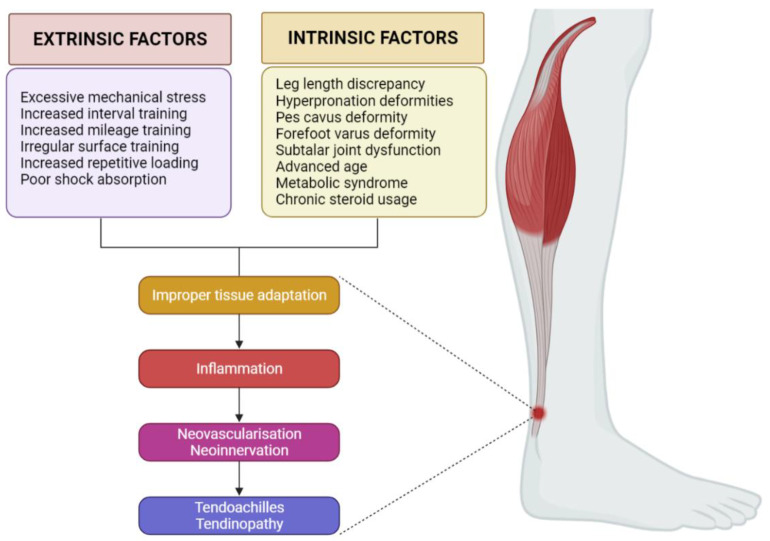
Pathogenesis of Achilles tendinopathy. Created with BioRender.com (accessed on 23 February 2022).

AT risk factors can be classified into intrinsic and extrinsic, and isolated or combined. Intrinsic factors encompass lower limb biomechanical irregularities. These include leg length discrepancy, hyper pronation, pes cavus (high arches), varus deformity of the forefoot, and impaired mobility of the subtalar joint [10]. Systemic conditions associated with advanced age, metabolic syndrome, and use of certain medications such as corticosteroids, also make up intrinsic factors [11]. Extrinsic factors comprise excessive mechanical stress and unhealthy training habits such as increased interval training, increased mileage, training on irregular surfaces, increased repetitive loading, poor shock absorption, and so forth [10]. The pathological mechanism in AT seems to be a result of a failed healing response and neurovascular ingrowth in response to injury as shown in Figure 1 [12].

### 2.1. Biomechanics

Tendon homeostasis is mainly regulated by mechanical loading and cellular activity which are governed by neuronal and cellular mediators. These mediators are produced either locally or remotely and then transported via blood circulation or nerve supply [13,14]. Adequate mechanical loading stimulates anabolic responses, especially the upregulation of collagen gene expression [15,16]. To elaborate, collagen synthesis reaches its maximum around 24 h after physical activity and can last up to 80 h. Conversely, excessive physical stress leads to the degradation of collagen proteins and, therefore, a more catabolic response. The timing of the catabolic peak, however, precedes the anabolic peak. Ultimately, the outcome is a net loss of collagen around the first 24–36 h after exercise, followed by a net gain [17]. This means that adequate rest between physical activity sessions is essential for tissue adaptation. Maintaining a healthy tissue status by avoiding a predominant catabolic microenvironment may reduce potential risks of injury.

### 2.2. Inflammation

Excessive or repetitive loading puts the tenocytes under a lot of stress, which subsequently causes them to produce inflammatory molecules. This could therefore fragilize the collagen fibrils and render them more susceptible to microdamage [18]. For instance, elevated levels of inflammatory cytokines such as prostaglandin E2 (PGE2) have been found in tendons subjected to repetitive mechanical loading [19]. Animal studies found that injections of PGE2 into the tendon substance causes degenerative alterations in rabbits [20] whereas peritendinous injections of PGE1 lead to tendinopathy on histological examination of murine samples [21]. Numerous pro-inflammatory cytokines such as interleukins (IL)-18, -15, -6, -1β, and tumor necrosis factor-alpha (TNF-α) have also been detected in this disorder [22,23].

Schubert et al. demonstrated that granulation alteration of capillary vessels and inflammatory infiltrates consisting of macrophages, mast cells, and B and T lymphocytes can be found in AT [24]. This suggests that the innate immune system exerts a regulatory role in early tendinopathy. Indeed, in the acute phase of inflammation, the aforementioned cytokines are produced by many immune cells including lymphocytes, monocytes, and endothelial cells [25].

**Figure 2:**
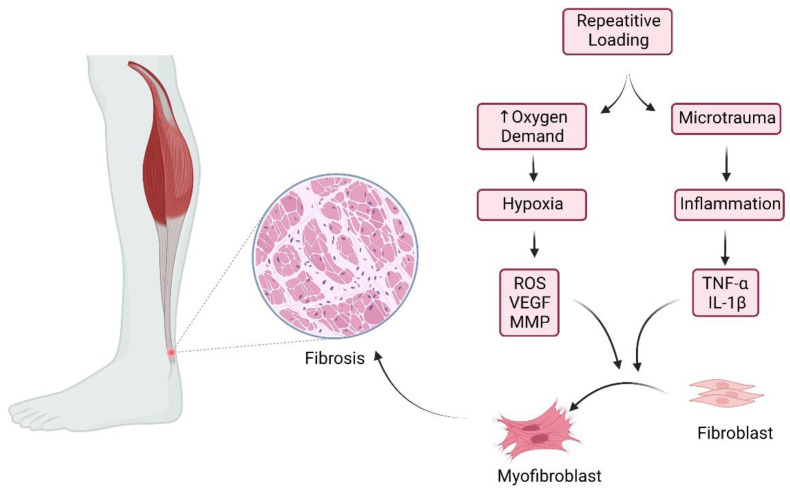
Pathophysiology of fibrosis in chronic Achilles tendinopathy. Created with BioRender.com (accessed on 23 February 2022).

The participation of macrophages during inflammation and tissue repair is crucial. Signaling pathways may stimulate the polarization of macrophages into either M1 (pro-inflammatory) or M2 (anti-inflammatory) subtypes [26,27]. In fact, numerous signaling molecules such as interferons, NF-κB, and glucocorticoid receptor activation pathways dictate the fate of monocyte differentiation and macrophage polarization [27]. That being so, inflammatory pathways in tendinopathy may influence macrophage polarization, leading to failed, fibrotic healing responses [28]. When subjected to repetitive mechanical stress, tenocytes, and fibroblasts combined with signals from the transforming growth factor β (TGF-β) and pro-inflammatory cytokines can differentiate into myofibroblasts [29]. These cells exert vital roles in tendon healing and tissue adaptation. Once the healing cascade ends, the mechanical stress on myofibroblasts is released, and these undergo programmed cell death, also known as apoptosis. However, should this mechanism fail, myofibroblasts trigger a hyperproliferative process, resulting in fibrosis, a clearly outlined histological feature of tendinopathy as shown in Figure 2 [30].

### 2.3. Neovascularization and Neoinnervation

It is known that VEGF upregulates angiogenesis and significantly contributes to the healing cascade by stimulating endothelial cell migration via chemotaxis and vasodilation. However, neovascularization in tendinopathy induces deterioration of the mechanical properties of the tendon and may contribute to rupture. Blood vessels themselves do not seem to be the cause of pain. Evidence indicates that the sprouting and ingrowth of sensory nerve fibers may follow the neoangiogenic process into the tendon proper in patients with tendinopathy, and then trigger nociception [24]. A solid association between the neuronal and vascular systems exists, where substance P and nerve growth factor (NGF) stimulate angiogenesis. Substance P induces angiogenesis by stimulating endothelial cell proliferation whereas NGF partially contributes to neovascularization by stimulating VEGF synthesis [31,32]. The ingrowth of sensory nerve fibers into the tendon proper is a characteristic feature of tendinopathy and occurs in regular tendon repair. This process is associated with nociception, followed by retraction correlated with decreased nociception once the healing process comes to an end [33]. Conversely, in tendinopathy, these nerves do not retract and the uncontrolled aberrant sprouting of the sensory nerve fibers indicates a failed healing response, leading to increased pain signaling. Additionally, this may also contribute to the hyperproliferative alterations featured in tendinosis [30].

### 2.4. Mechanical Loading and Unloading of the Tendon

The tendons are constantly exposed to mechanical forces throughout life. In adequate intensity, as seen in physical activity, mechanical loading can produce numerous health benefits for not only for tendons but the whole musculoskeletal system as well [19]. These effects include increases in cross-sectional area, tendon stiffness, and tensile strength [19]. These positive effects arise as a consequence of the anabolic responses generated by tendon cells, especially increases in the synthesis of collagen type I in the peritendinous tissue [34]. Another major biological response that is generated by loading is the expression of the Eb isoform of insulin-like growth factor 1 (IGF-1), sometimes referred to as mechano-growth factor (MGF) [35]. This anabolic growth hormone plays a key role in tendon biology as it translated the mechanical forces into biochemical signals in order to induce biological changes.

When mechanical forces cease, tendons relax and the constant production of signals ends. An in vitro study revealed that a few days after removal of mechanical loading there is a downregulation of mRNA for tenomodulin and collagen, which is accompanied by disorganization of the aligned fibrils in the tendon structure [36]. Interestingly, administration of transforming growth factor beta (TGF-β) to these cells does not reverse downregulation, illustrating the influence of physical tension on phenotypical alterations of tendons [36]. In vivo, the circumstances are obviously different, but generally, immobilization of lower limbs will lead to reduced collagen synthesis rates by approximately 80% [37]. Moreover, another study showed that 2 weeks of immobilization downregulates LOX and scleraxis whilst upregulating matrix metalloproteinase-2 (MMP-2) in human patellar tendon [38]. This suggests that inactivity favors reduced collagen synthesis and signaling stimulation as well as accelerated proteolytic activity.

## 3. Management of AT

Initial treatment usually employs a multifactorial approach envisioning a combination of rest, administration of analgesics, orthotic treatment such as heel lifts, physical rehabilitation primarily focused on eccentric exercises, photo-biomodulation therapy, nutritional supplements, friction massage, and dry needling [39]. The main problem associated with conservative treatments is that often these techniques only target nociception but do not address the pathophysiological processes of the disease itself [40]. Conservative treatments may work well for some cases of mild injuries, but this may not be the case when a patient presents with severe musculoskeletal damage. It is estimated that in approximately 25–30% of patients with chronic AT pain, non-surgical treatment is not successful and surgical treatment may be needed [41]. Therefore, when conservative treatment fails, physicians may consider a surgical alternative. The frequency of AT surgery has been shown to increase with the patient’s age, duration of symptoms, and occurrence of tendinopathic changes [41].

Although surgeries may become ultimately inevitable for patients with more long-lasting or aggressive AT conditions, it is worth noting that these alternatives are often accompanied by risks [42]. Surgical techniques may include both open and percutaneous procedures for removal of the injured tissue or stimulate repair by longitudinal incisions. Rarely, irreparable tendon damage may need to be replaced by tendon transfer or grafts.

Authors have been raising questions about the success rates of open surgery for chronic AT over the last 50 years. In comparison to minimally invasive techniques, open procedures were not different in success rates. However, there was still a tendency for more complications in the open procedures [43]. To circumvent these hurdles, medical experts have been shifting their focus towards orthobiologics, which are novel therapeutic tools that continue to expand and reveal promising results in both pre-clinical and clinical research [44].

## 4. Orthobiologics in AT

### 4.1. Cellular Therapy in AT

**Figure 3:**
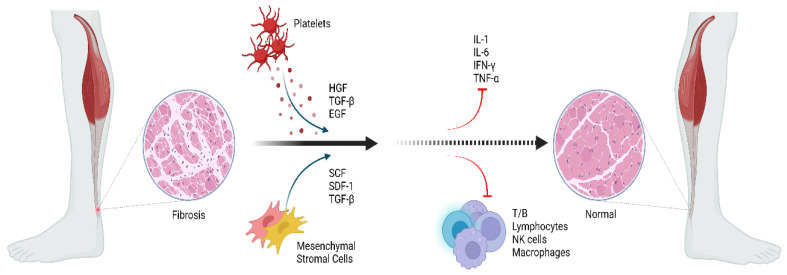
Potential mechanism of action of orthobiologics in Achilles tendinopathy. Created with BioRender.com (accessed on 23 February 2022).

By standard definition, orthobiologics are products derived from substances naturally found in the body that can expedite and improve the healing process of an orthopedic injury [45]. The most widely cited examples include embryonic stem cells, mesenchymal stromal/stem cells, and induced pluripotent stem cells [5,46]. This array of orthobiologics has enabled researchers to target different diseases at a cellular level and has bestowed physicians with growing potential in the ever-expanding field of regenerative medicine [47]. The mechanism of action of orthobiologics in Achilles tendinopathy is depicted in Figure 3.

#### 4.1.1. Embryonic Stem Cells (ESCs) and Induced Pluripotent Stem Cells (iPSCs)

Embryonic stem cells (ESCs) are isolated from the inner cell mass of the blastocyst during the pre-implantation period. Two of the most refined properties of these cells are their capacity to infinitely proliferate without differentiation and, at the same time, retain the potential to generate all three germ layers [48]. These properties are, respectively, termed self-renewal and pluripotency.

The tenogenic potential of ESCs has been successfully demonstrated both in vitro and in animal models, but the transition to clinical trials still requires further investigation of their tumorigenic potential [49,50,51]. In a large animal model trial, ESCs showed improved healing in collagenase-induced flexor tendinitis [52]. Tissue architecture, tendon size, tendon lesion size, and fiber patterns of the tendons were significantly improved on histologic sections and ultrasound in the horse-treated group, even without in vitro pre-differentiation.

On the other hand, how to differentiate ESC into tendon lineage is the key point. Some authors have suggested that a stepwise differentiation of human ESCs (hESC) into MSCs could potentially allow these multipotent cells to form tendon-like tissues, with the advantage of avoiding teratoma formation [53]. In vitro and in vivo studies showed that ESC-differentiated MSCs (hESC-MSCs) markedly presented tenocyte-like morphology and expressed tenocyte-related gene markers such as COL-1 and -3 and SCX [54]. Additionally, tendon repair treated with hESC-MSCs showed better ectopic tendon regeneration and mechanical properties than did controls, with hESC-MSCs remaining viable for longer periods [54].

Similar results were shown by the use of hESC-MSCs incorporated within collagen sponge scaffold to promote tendon regeneration [55]. hESC-MSCs exhibited tenocyte-like morphology and also positively presented tendon gene markers (COL-1 and -3, Epha4, and Scleraxis). After in vivo transplantation and under the mechanical stimulus, the tissue displayed better alignment and configuration of the collagen fibers and superior mechanical characteristics [55]. Other potential benefits of hESC-MSCs transplantation in tendon pathology is the in situ environment-modifying effects, indirectly favoring tissue regeneration. However, the use of ESCs may still be limited by ethical concerns since a sacrificed embryo is needed [56,57,58,59].

In this sense, the later discovery of Induced Pluripotent Stem Cells (iPSCs) can potentially solve the ethical problem of ESCs, in which researchers generated iPSCs from totally differentiated cells through specifically transcription factors delivered by retroviruses or by miRNA delivered directly to generate integration-free iPSCs [60,61]. Human iPSCs have been shown to better repair rat patellar tendon window defects in comparison to non-iPSCs treated tendons, demonstrating by macroscopical, histological, and biomechanical analysis that human iPSC promotes tendon repair in animal models [62]. Tenocytes derived from human iPSC can provide a therapeutic option for tendon injury. In the study, iPSC-tenocyte grafting contributed to motor function recovery after Achilles tendon injury in rat models via paracrine effect and engraftment. [63] While the use of ESCs and iPSCs is progressively growing, it has not been reported in human studies of tendon tissue engineering yet. On the other hand, recent progress in culturing these cells to properly differentiate them brings great expectations for the use of human-ESC based therapy in the near future [64,65].

#### 4.1.2. Mesenchymal Stromal Cells (MSCs)

MSCs possess self-renewal potential and have the ability to differentiate into specific mature cell lineages [66]. They are characterized by a set of specific cell surface cluster differentiation markers (CD), which are known to express a range of cell-lineage-specific antigens which differ depending on culture preparation, duration, or plating density [67,68]. MSCs can manipulate and control their local microenvironment due to the paracrine and autocrine effects [69]. MSCs do not trigger aggressive immunogenic episodes and they can be easily isolated, facilitating allogenic transplantation in appropriate circumstances. Therefore, these cells may be considered immune evasive, however, the regenerative effects of MSCs in cellular-based therapies are usually more associated with their homing and engraftment abilities in target tissues [70]. Furthermore, MSCs have a rather short life span and are ultimately phagocytized by monocytes, subsequently stimulating the production of T-regulatory (Treg) cells, thereby maintaining homeostasis and self-tolerance [71,72]. MSCs act as trophic mediators to attenuate escalated apoptosis, fibrosis, and inflammation whilst stimulating cell proliferation and differentiation via paracrine and autocrine signaling [73].

**Bone marrow-derived MSCs (BM-MSCs):** The cellular components of bone marrow can be divided into non-hematopoietic cells (pericytes, endothelial cells, osteoblasts, adipocytes, and Schwann cells) and hematopoietic cells (neutrophils, lymphocytes, megakaryocytes, monocytes, and osteoclasts) [74]. Additionally, there is also the presence of the hematopoietic stem cells (HSC) and mesenchymal stromal/stem cells (MSCs), the two major adult stem cell types found in this tissue. The MSCs present in bone marrow contain a potent anti-inflammatory cytokine known as interleukin-1 receptor antagonist (IL-1Ra) [75]. IL-1Ra also reduces matrix degradation, MMP-3, and TNF-α gene expression, PGE2 secretion, chondrocyte apoptosis, and enhances collagen deposition. [76] Collectively, the effects elicited by IL-1Ra are of great clinical value as they can bring significant pain alleviation to the patient and improve the state of prolonged tissue injury inflammation, especially in tendinopathies as shown in Figure 3. Intratendinous administration of BM-MSCs in rabbit model AT improves biomechanical (improved biomechanical modulus) and histological (improved collagen fibers organization) scores in the early phase of tendon healing [77]. In a rat model of Achilles tendon rupture, BM-MSCs show superior tendon healing potential than PRP in terms of histological, biochemical, and immunohistochemical scores [78]. In a rat model of Achilles tendon rupture, an increased expression of Tenascin-C was observed equally in the groups treated with tendon stem cells (TSCs) and BM-MSCs but TSCs exhibited higher regenerative potential than BM-MSCs. Hence, TSCs are the better sources of stem cells for tendon regeneration [79]. Under ultrasound (USG) guidance, autologous bone marrow aspirate concentrate (BMAC) injected intralesionally into the mid substance tendinopathic region of Achilles in a female patient with chronic MRI confirmed AT exhibited less pain with the normal activity of daily living (ADL) after 2 months of post-intervention. Improvement in relative strength intensity was observed in T1W-MRI images after 10 weeks post-intervention [80]. Improved Achilles tendon rupture scores were observed with autologous BMAC augmentation in Achilles tendon rupture [81]. BM-MSCs promote early rehabilitation, lower incidence of re-rupture, improvement of pain scores, and amelioration of tendon structure and strength, without the occurrence of serious complications [81,82,83,84,85,86,87]. van den Boom et al. derived level 4 evidence for BMAC augmentation in Achilles tendon repair in terms of improved PROMs and absence of re-tears in 2.5 years follow-up [88]. However, more robust data are still required to further support the efficacy and safety regarding the clinical administration of BMA for AT, more specifically.

**Adipose tissue-derived MSCs (AD-MSCs):** In recent years, adipose tissue and its derivatives have also received a considerable amount of attention from the scientific community by presenting itself as a novel and potential cell source for tissue engineering and regenerative medicine [89,90]. A total yield of stem cells in adipose tissue was approximately 40 times greater than bone marrow [91,92,93]. SVF, a product of adipose tissue, carries a wide variety of cells, including endothelial cells, preadipocytes, type 2 macrophages, T cells, pericytes as well as mesenchymal stromal/stem and progenitor cells [89,90,94]. The application of SVF for the treatment of tendinopathies, specifically, yields satisfactory regenerative outcomes [95,96,97,98]. An in vivo study with SVF and AD-MSCs demonstrates the maintenance and induction of tendon fiber organization [96]. Usuelli et al. demonstrated that the intratendinous SVF injection exhibited faster recovery results at just 15 days after treatment for AT [95]. Piccionello et al. revealed the significant findings in the ovine model of tendinopathy [96]. Matrix composition and collagen deposits in treated tendons are significantly enriched, and neo-angiogenesis is improved within the lesion sites and it was concluded that the reorganization of tendon fibers is just as important as the proliferation, differentiation, and immunomodulatory capacities of SVF cells [96]. In a collagenase-induced AT mice model, AD-MSCs facilitate neovasculogenesis, upregulate tendon repair, downregulate ectopic ossification, and inhibit inflammation in Achilles tendon healing [99]. At 3 months follow-up, the improvement of AOFAS and FADI scores was observed in surgically managed Achilles tendon tears with micro fragmented adipose tissue (M-FAT) [100]. M-FAT downregulates the expression of type 3 collagen and metalloproteases-1 in a significant manner and upregulates the production of VEGF, IL-1Ra, and IL-6 in an in vitro model of tendinosis [101]. Tenogenically differentiated AD-MSCs upregulate the gene expression of COL-1 and -3, scleraxis, decorin, tenascin-C, and tenomodulin, modulate cytoarchitecture, and improve the histological score, organization of collagen fibers, recovery of elastic modulus, and tensile load of tendons over time in Achilles tendon repair in vivo [98]. SVF derived from adipose tissue pose superior results in terms of clinical and functional outcome in AT when compared with PRP [102,103]. van den Boom et al. derived level 3 evidence for allogenic AD-MSCs in the management of AT when compared with PRP [88]. Although the basic science studies in the literature have already revealed positive outcomes, there is still a great need for more robust clinical data to further validate the efficacy and safety of the application of adipose tissue-derived products in tendon healing.

### 4.2. Acellular Therapy in Achilles Tendinopathy

Acellular therapy marks the application of nanomedicine principles in the management of musculoskeletal disorders. The micromolecules from cells and tissues play a significant role in targeting the desired site with therapeutic applications. The most commonly used acellular biological products are platelet-rich plasma (PRP), concentrated growth factors, and exosomes from various sources.

#### 4.2.1. Platelet-Rich Plasma (PRP)

Chronic tendinopathy creates a pro-inflammatory environment and hinders the healing cascade due to precarious blood supply and comparably slower cell turnover in the case of tendons [104]. Literature evidence supports that PRP has targeted therapeutic applications in musculoskeletal disorders by enhancing regeneration of diseased or degenerated tissues. By concentrating platelets, the growth factors are released from alpha granules of platelets which enhance the natural healing cascade. PRP contains WBCs and chemokines, which regulate inflammatory responses [105,106]. The type of PRP to be used depends upon the disease condition and targeted site in the body to have maximum benefits outweighing the risks. Yoshida et al. [107] demonstrated that the combination of leucocytes with platelets in an ACL fibroblast culture promoted significant increases in type I and type III pro-collagen gene expression, collagen production, and cellular proliferation. The administration of PRP accelerates and hastens neovascularization and stimulates the potentiation of the resident stem cells and the subsequent restoration of injured tissue. Upon activation of PRP, numerous pockets of growth factors and cytokines release in the desired site and exert anabolic and anti-inflammatory actions by potentiating various cells and their secretomes as shown in Figure 3 [108]. PRP potentiated the differentiation of TSCs to mature tenocyte by increasing the proliferation and collagen production [109]. The platelets in PRP stimulate macrophages and fibroblasts to repair the damaged collagen fibrils of the tendon and enhance neovasculogenesis and collagen organization in the injured tendon [110,111].

The outcome of PRP injection for AT demonstrated decreased vascularity and changes in the tendon thickness as reported in a few studies [112,113,114,115] whereas a few researchers have provided controversial data stating the increased tendon thickness after 3 months follow-up [116,117]. Filardo et al. demonstrated a stable outcome to a medium-term follow-up with repeated intra-tendinous PRP injection in recalcitrant AT [118]. Gaweda et al. reported a significant decrease in tendon thickness and hypoechoic lesions along with the normalization of peritendineum in AT when treated with PRP [119]. Deans et al. demonstrated a significant clinical improvement in recalcitrant AT with a single dose of autologous conditioned serum along with regular exercises and therapeutic ultrasonography [120]. Monto et al. reported significant USG and MRI changes in Achilles tendon substance in pre-and post-PRP treatment in AT [121]. de Vos et al. reported no greater improvement in pain and activity, when AT is treated with eccentric exercises and PRP [113]. In a meta-analysis with seven clinical trials by Chen et al., no trial has demonstrated that PRP improves either clinical or functional outcomes in AT and hence RCTs were expected to test the hypothesis [122]. In a meta-analysis, Liu et al. reported limited evidence support that PRP is not a superior treatment to placebo management in chronic AT [123]. Zhang et al. reported no improvement in VISA-A scores, tendon thickness, or color Doppler activity in AT with PRP [124], whereas Madhi et al. demonstrated a significant improvement in VISA-A scores with the usage of PRP in AT [125]. PRP is found efficacious in young to middle-aged patients with non-insertional AT compared to old aged patients. This data is ascribed to biomechanical differences in the tendinous substances [126]. Townsend et al. formulated post-PRP protocol for AT by the initiation of stretching exercises by 2 weeks after injection and then full return to play was advised after 6 weeks of PRP injection [127]. Despite controversy in the literature, many studies share a common ground in the sense that PRP consistently presents itself as a safe and effective biological agent for the amelioration of both chronic and acute Achilles tendon injuries, with significant improvements in pain and functional outcomes [112,123,128,129].

#### 4.2.2. Exosomes

The sourcing of exosomes (Exos) presents a major challenge in scaling production in terms of commercialization and therapeutic efficacy of clinical applications. Exos can be derived from either cellular (hematopoietic cells, mesenchymal stromal cells, immune cells and tissues in the form of organs) or noncellular sources (body fluids). Production of large amounts of Exos is expensive, technically demanding, and ethically challenging. Local administration of Exos in the bone–tendon interface downregulates the genes responsible for pro-inflammatory cytokines, excessive scar formation, cellular apoptosis, and M1 macrophages and upregulates the genes responsible for anti-inflammatory cytokines and ECM synthesis [130,131]. MSC-Exos modulate collagen organization and macrophage polarization, proliferate tenocyte and fibroblast cells, and inhibit tenocyte adhesions in tendon disorders [132]. BM-MSC-derived Exos enhance the healing of the tendon–bone interface by regulating M2 macrophage polarization [133]. Administration of TSC-derived Exos into rat AT leads to downregulation of MMP-3 gene expression with upregulation of TIMP-3 and COL1A1 gene expression, balancing of ECM in the tendon, and potentiates ethnogenesis of TSCs [134]. TSCs regulate immunomodulation in tendons through c-Jun N-terminal kinase and STAT-3 signaling [134]. TSC-derived Exos containing TGF-β enhance the migration, proliferation, and differentiation of TSCs through Smad2/3 and ERK1/2 signaling pathways [135]. Tenocyte-derived Exos express higher levels of CD-9 and -61, TSG-101, COL-1 and -3, TNMD, Shc, p-ERK1/2, and integrin β1 to enhance the proliferation of MSCs [136].

Transplantation of BM-MSC-derived EVs promotes tendon healing in rat model AT in a dose-dependent manner with the organization of tendon fibers, architecture, and type 1 collagen [137]. AD-MSC-derived Exos enhance tenogenic differentiation of TSCs and reduce inflammation through SMAD2/3 and SMAD1/5/9 signaling pathways [138]. AD-MSC-derived Exos enhance the proliferation of CD146^+^ TSCs. An in vitro study demonstrated a dose-dependent tenocyte proliferation and migration when administered with TSC-Exos through PI3K/AKT and MAPK/ERK1/2 signaling pathways [139]. Modified nitric oxide nanomotor-derived Exos loaded with microneedle downregulate the pro-inflammatory cytokines and upregulate anti-inflammatory cytokines with enhanced expression of COL1A1 and prevented ECM degradation in AT [140]. Polarized M2 macrophage-derived Exos facilitate peritendinous fibrosis of Achilles tendon injury by upregulating the expression of type 1 and 3 collagen, α-SMA, and TGF-β1, via the MiR-15b-5p/FGF-1/7/9 pathway by delivery of circRNA-Ep400 [141]. Macrophage-derived Exos induce peritendinous fibrosis after tendon injury through the miR-21-5p/Smad7 pathway [142]. Supplementation of collagen with purified exosome product demonstrated intrinsic healing in tendon disorder. Application of exosomes for tendon injury show less microscopic and macroscopic circumferential adhesions [143]. BM-MSC-derived exosomes loaded with fibrin glue enhance the expression of tenomodulin and type 1 collagen, improve the biomechanical properties of neotendon, and facilitate the differentiation and proliferation of resident stem cells into local TSCs in vivo [144].

## 5. Future Prospects in AT

The concept of “Smart Exosomes” describes the manipulation, programming, and re-programming of the exosome and secretome contents to facilitate the management of tendon injury [145]. These smart exosomes act in the bone–tendon interface to regulate the pro-inflammatory to the anti-inflammatory environment, improve the biomechanical properties, and enhance proliferation, migration, and differentiation of tenocytes [145].

Engineered tenogenesis involves the mixture of stem cells and growth factors loaded onto scaffolds to accelerate tendon regeneration and modulate Young’s modulus of the tendon. Yang et al. developed engineered tenogenesis TSCs admixed with scaffolds such as chitosan/β-glycerophosphate/collagen hydrogel in a rat AT model [146]. Autologous BM-MSCs loaded with collagen sponge in a vicryl mesh tube exhibited organized collagen fibers, neovasculogenesis, and spindle-shaped tenocytes with complete regeneration of tendons in a rabbit model Achilles tendon rupture [147]. In a rat model of Achilles tendon defects, seeding of hypoxia preconditioned AD-MSCs on small intestine submucosa (SIS) reported superior healing than cell-free SIS [148]. Tendon engineering with TSCs and scaffolds increases the expression of type 1 collagen and SCX genes [149]. Engineered tendon matrix from TSC potentiates the formation of tendon-like tissue and matrix, which helps to repair the tendon-related disorders in vivo [150]. In rat patellar tendinopathy, SCX gene transduced TSCs promote tendinogenesis when compared to mock-transducer cells [151]. miRNA engineered umbilical cord-MSC derived Exos are directed towards tendon healing by mTOR/TGF-β1 signaling to deliver miR-29a-39 [152]. In a rat model TA, the administration of conditioned medium of TSCs induced with HGF enhances tendon healing in TA by upregulating the migration of tendon fibroblasts, modulating ECM composition, and improving the biomechanical property of Achilles and organization of collagen fibers. [153] Scaffold-free Scleraxis-programmed tendon progenitors enhance the repair of full-size Achilles tendon rupture [154].

“Extracellular vesicles-educated macrophages” (EEMs) were generated by exposing CD14+ macrophages to EVs, which were directed towards tendon healing [155]. These EEMs are (a) generated using off-the-shelf EVs and monocytes from autologous blood, (b) terminally differentiated cells, and (c) used to treat inflammatory disorders. EEMs accelerate vasculogenesis by upregulating endothelial cell count, downregulating the M1/M2 ratio, and improving biomechanical properties during Achilles tendon healing [155]. The delivery of TNF-α primed MSCs via 3D PLG scaffold modulated macrophage polarization and cytokine production to further accentuate the more regenerative MSC-induced healing response in Achilles tendon defects [156].

The primary obstacle in clinical translation of these advanced cellular therapies remains in the lack of evidence of their effectiveness in the sufficient animal models of chronic nature to simulate Achilles tendinopathy since most of the animal models analyzed the augmented healing in acutely injured tendons [157]. Moreover, it is also important to standardize the isolation procedures of these biological therapies before embarking on their clinical translation [158]. Hence, translation of these biological therapies requires the combined effort of the surgeons to define the clinical problem along with the limitations of the conventional treatment strategies, product development experts to determine the potential of the market and identify the key inadequacies in the current treatment methods, biologists, materials scientists, and designers to design the implant to achieve the desired biological effect with sufficient material of appropriate specification to ensure enhanced functionality [159].

## 6. Conclusions

Cellular and acellular therapies have shown promising in vitro and in vivo results in the management of Achilles tendinopathy in laboratory and animal models which provides sufficient evidence to initiate clinical trials. However, our knowledge on the biology and application of PRP, MSCs, and MSC-based EVs is still evolving, hence further studies clarifying the comprehensive therapeutic mechanisms of these cellular or acellular-based therapies remain crucial to develop the best treatment regimen for AT. Moreover, clinical translation of these biological therapies requires standardization of their isolation procedures as a necessary prerequisite, which remains as lacunae, to be addressed soon.

## Data Availability

Not applicable.
